# Why too soon? Predictors of time to diabetic peripheral neuropathy among newly diagnosed diabetes mellitus patients: a multicenter follow-up study at health-care setting of Ethiopia

**DOI:** 10.1186/s13690-023-01202-3

**Published:** 2023-10-21

**Authors:** Gebiso Roba Debele, Samuel Abdisa Kuse, Bilisumamulifna Tefera Kefeni, Abdi Geda, Wakuma Wakene Jifar, Keno Melkamu Kitila, Mohammedamin Hajure

**Affiliations:** 1 Department of Public Health, College of Health Sciences, Mattu University, Mattu, Ethiopia; 2 Department of Midwifery, College of Health Sciences, Oda Bultum University, Chiro, Ethiopia; 3 Department of Pharmacology, College of Health Sciences, Mattu University, Mattu, Ethiopia; 4 Department of Psychiatry, College of Health Sciences, Mattu University, Mattu, Ethiopia

**Keywords:** Incidence, Diabetic peripheral neuropathy, Predictors, Diabetes mellitus

## Abstract

**Background:**

Due to the rising number of diabetic patients, the burden of diabetic peripheral neuropathy (DPN) is clearly posing a major challenge to the long-term viability of the health-care system. Despite this, most DPN epidemiological research in eastern Africa, including Ethiopia, has so far been limited to survey studies. Thus, we determined the incidence of DPN and its predictors among diabetic patients in tertiary health-care setting of southwest Ethiopia.

**Methods:**

A multicenter retrospective follow-up study was carried out on 567 randomly selected diabetic patients. Data were entered using Epi-Data v4.6 and analyzed using R v4.0.4. The survival curves were estimated using the Kaplan-Meier, and compared using Log-rank test between groups of categorical variables. The PHA were evaluated using the Schoenfeld residuals test. Multivariable Gompertz proportional hazard model was used to examine the predictors of DPN at 5% level of significance.

**Results:**

Overall, of 567 DM patients 119 developed DPN with an incidence rate of 3.75, 95%CI [3.13, 4.49] per 100 PY. About 15.13% and 69% of DPN cases occurred within 2 and 5 years of DM diagnosis, respectively. In the multivariable Gompertz PH model, being female [AHR = 1.47; 95% CI (1.01, 2.15)], T2DM [AHR = 3.49 95% CI (1.82, 6.71)], having diabetic retinopathy [AHR = 1.9 95% CI (1.25, 2.91)], positive proteinuria [AHR = 2.22 95% CI (1.35, 3.65)], being obese [AHR = 3.94 95% CI (1.2, 12.89)] and overweight [AHR = 3.34 95% CI (1.09, 10.25)] significantly predicts the future risk of DPN.

**Conclusion:**

Nearly, 7 in 10 of DPN cases occurred within short period of time (5 year) of DM diagnosis. Being female, T2DM, DR, positive proteinuria, obese and overweight significantly predicts the risk of DPN. Therefore, we recommend screening and early diagnosis of diabetes with its complication. While doing so, attention should be given for DM patients with DR and positive proteinuria at baseline.

**Table:** 

**Text box 1. Contributions to the literature**
• Given the increasing prevalence of T2DM, its complications, particularly DPN, continue to present serious challenges to healthcare system in developing countries.
• Despite this fact, there are few studies, and the literature that is currently available is limited to survey studies in Ethiopia.
• Furthermore, to our knowledge, no previous investigation of DPN has used advanced survival analysis (Gompertz survival analysis).
• The Identification of predictive factors and timing of DPN development would enable early detection of the condition, reducing the financial loss related to complications like diabetic foot ulcers and amputation brought on by DPN.

## Introduction

Despite the fact that diabetes mellitus (DM) has been the focus of most international organizations, the disease’s burden and complications remain a global issue [1]. According to the 2021 international diabetic federation (IDF), there were an estimated 537 million people living with DM and 6.7 million deaths due DM and its complications which corresponds to one death for every five seconds [2]. Because of its complications, such as Diabetic peripheral neuropathy (DPN), DM is a serious condition [3]. DPN is one the most common vascular complication of DM and is estimated to affect around half of the people with diabetes [4–6]. One prospective cohort study revealed an increase of DPN after five years from 13.2 to 34.2%, representing a 2.6-fold increase [7].

Clinically, DPN is defined as the presence of symptoms or signs of peripheral nerve dysfunction in people with diabetes after other possible causes have been excluded [8]. DPN mainly alters the symmetrical sensory function causing abnormal feelings and numbness [9]. Studies showed that the prevalence of DPN ranges from 6 to 87% in developed countries [10–12] and 46% pooled prevalence in Africa with a higher proportion in West (49.4%) and Central (35.9%) Africa [13]. In Ethiopia, 32.8% of DM patients develop diabetes-related complications [14] with the prevalence of DPN ranging from 29.5 to 52.2% [15]. Other study found the pooled prevalence of 22% in Ethiopia, with a higher proportion in Oromia 27%, and 16% in southern nation nationalities and peoples region [16].

DPN is a major cause of disability, leading to foot ulcers, amputations, urinary inconsistence and sexual dysfunction [17]. For example DPN raises the global burden of diabetic foot ulcers, with a rate of 7.2% in Africa [18]. Similarly, 25–90% of amputations are thought to be attributable to the combination of DPN and other infection due to diabetes [19]. Not only the above consequences, painful DPN which affects 20–30% of patients, has been linked to severe physical and psychological distress leading to poor patients quality of life [20]. All these consequences of DPN will unavoidably end in the reduction in the quality of life and significant economic burden both to the patients and society.

Several epidemiological researches around the world identified older age [21–23], smoking [22, 24], high low density lipoprotein cholesterol (LDL-C), triglycerides, and lower high density lipoprotein cholesterol (HDL-C) [21, 22] as a risk factors for DPN. Dyslipidemia [23, 25], the presence of cardiovascular disease (CVD), diabetic retinopathy (DR) was also significantly associated with DPN [24, 25]. Other studies found that, female gender were the strongest predictors followed by longer duration of DM [23–26]. Higher levels of Body Mass Index (BMI) at baseline were also significantly increases the risk of incident DPN [21].

DPN is poorly managed because of its insidious onset and delayed diagnosis [27]. The identification of predictive factors and the time when DPN occurs after DM diagnosis would facilitate early screening for DPN and DPN-related symptoms. Physicians’ care for patients with DPN might also benefit from a greater understanding of the factors that predict DPN. Despite this fact most of previous studies in Ethiopia focused on survey data using crossectional study design which cannot solve the above problems. Thus, the current study determined the incidence of diabetic peripheral neuropathy and its predictors among diabetic patients in tertiary health-care setting of southwest Ethiopia.

## Methods

### Study design, periods and settings

The current study is a, retrospective follow up study done at Jimma University Medical center (JUMC) and Mettu Karl Comprehensive and Specialized Hospital (MKCSH) to assess the incidence DPN and its predictors among T2DM patients from September 9, 2013 to February march, 2021. JUMC and MKCSH are the largest tertiary health-care setting that found in Jimma zone and Ilu Ababor zone respectively, Oromia regional state, southwest Ethiopia. JUMC is found at about 352 KM and MKCSH at 600KM from Addis Ababa, the capital city of Ethiopia. In both hospitals, follows-up service for chronic disease is being provided and diabetic clinic is found separated from other chronic disease.

### Population

The source population for the current study was all type 1 DM (T1DM) and type 2 DM (T2DM) patients (age of 15 and above years old) who had follow-up at JUMC and MKCSH. We included all newly diagnosed with T1DM and T2DM patients during the follow-up visits from September 2013 to August 2015 at JUMC and MKCSH and those patients were followed until March 2021. Once diagnosed for DM, follow up was started immediately for all patient. Diabetic patients with DPN at the date of diagnosis and no baseline records at baseline were excluded from the study. In addition, diabetic patients with unknown date of DM and DPN diagnosis were excluded from the study.

### Sample size and sampling procedure

**Fig. 1:**
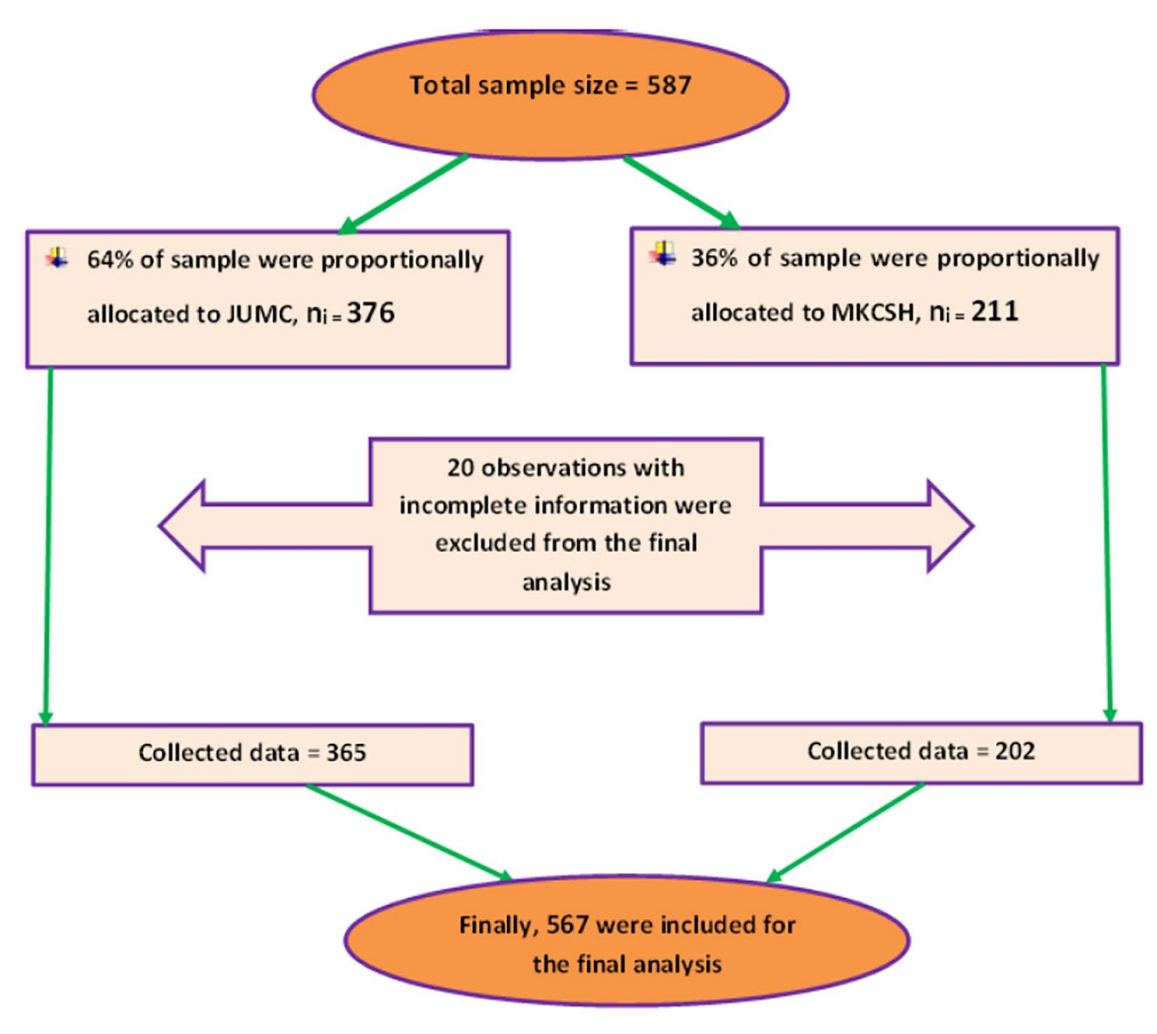
Schematic framework of the sampling techniques for selection of the study subjects

The final sample size was determined based on the power method by using the Schoenfeld formula using Stata version 14 [28]. We assumed 80% of power, 10% of withdrawal probability and 95% confidence level. Considering 1.82 adjusted hazard ratio (AHR) of other DM complication and 16.6% probability of events from previous study done in Ethiopia [29] the final sample size was calculated to be **587**. Then subjects were selected using simple random sampling technique after proportionally allocated to both hospital (Fig. 1) by collecting the identification number of DM patients from the registration book.

### Measurement of variables and operational definition

The primary outcome of the current study was time to DPN and the event of interest was the development of DPN within the follow-up period. DPN can be either small fiber or large fiber neuropathy. Pain, tingling, and paraesthesia are symptoms of small fiber neuropathy, which diagnosed with a pinprick and temperature examination. Numb feet and gait ataxia are symptoms of large fiber neuropathy, which are confirmed by touch sensitivity with a 10 g monofilament, vibration sensibility with a biothesiometer, and ankle reflex [8, 30]. When a participant had more than one endpoint, the initial event was utilized to determine when DPN had started. This remark implies that a pinprick and temperature are used to identify small fiber DPN. While 10 g monofilament touch sensitivity, biothesiometer vibration sensitivity, and ankle reflex are used to diagnose big fiber DPN. Sociodemographic, clinical factors, and physiologic characteristics were among our independent variables. Time to DPN was defined by the time it takes between being diagnosed with DM to developing DPN. Patients who did not acquire diabetic neuropathy during the research period, or who died, lost follow-up, or transferred out before developing DPN were considered as censoring. DR is a microvascular consequence of diabetes that is diagnosed by ophthalmologists through clinical examination or indirect ophthalmoscopy [31]. Hypertension (HTN) is defined as a systolic/diastolic blood pressure of 140/90 mmHg or above on two or more separate days, with the measurement taken from a medical record review [32].

### Data collection and quality control

This secondary data was acquired from the study participants’ medical records by professional nurses using a pre-approved standardized checklist. The consistency and completeness of the checklist were checked. Any missing data was double-checked and clarified. From the time of enrolment (September 9, 2013) until the end of the study (March, 2021), we monitored the subjects retrospectively.

### Data processing and statistical analysis

Epi-Data version 4.6 was used to enter data, which was subsequently exported to R version 4.0.4 for further cleaning and analysis. The study population was described using descriptive statistics such as mean (standard deviation) normally distributed for continuous variables or median (inter-quartile range) for non-normally distributed continuous variables. Tables and frequencies with percent were used for categorical variables. The baseline categorical variables of the cohort were compared using the Pearson’s chi-square test based on the status of DPN during follow-up.

The number of new cases (DPN) per total original study population was used to determine cumulative incidence, and the incidence rate was derived as the number of new cases per patient-years at risk. The multivariable regression included a variable with p value less than 0.2 in bivariable analysis. The pseudo-Variance Inflation Factor (VIF) was used to check multicollinearity within independent variables. The survival curves were estimated using the Kaplan-Meier approach, and the Log-rank test was used to evaluate survival time between groups of categorical variables. The proportional hazard assumptions (PHA) were evaluated using the Schoenfeld residuals method and Akakie Information Criterion (AIC) was used to choose a parsimonious survival model. The model’s quality of fit was determined using the Cox Snell residual plot. Finally, the Gompertz proportional hazard model were fitted, and variables with a significance level of 0.05 were identified as a predictors of incident DPN.

## Results

### Socio-demographic and clinical characteristics

Of the initial cohort of 587 enrolled participants, 20 (3.75%) records with incomplete information were excluded. We included the remaining 567 participants in the final analysis. Male patients comprised 60.49% of the study’s participants, while urban inhabitants thought up 58.2%.

The baseline socio-demographic and clinical characteristics of study participants by incident DPN status are shown in Table 1. The number of incidents were higher in those who were older and took oral hypoglycemic medications more often at baseline. Those with nephropathy, Acute Kidney Injury (AKI), HTN, positive proteinuria, T2DM, family history of DM, or CVD at baseline also had a greater percent of incident DPN.

**Table 1:** Baseline socio-demographic and clinical characteristics of study participants by incident diabetic peripheral neuropathy status during of follow-up from 2013 to 2021

Variables	Incident Peripheral Neuropathy		Total (n = 567)	P-values
	No DPN (n = 448)	DPN (n = 119)		
Age				
< 65 year	401 (80.68)	96 (19.32)	497 (87.65)	0.009
>/=65 year	47 (67.14)	23 (32.86)	70 (12.35)	
Sex				
Male	279 (81.34)	64 (18.66)	343 (60.49)	0.092
Female	169 (79.01)	55 (20.99)	224 (39.51)	
**Residence**				
Urban	262 (79.39)	68 (20.61)	330 (58.20)	0.792
Rural	186 (78.48)	51 (21.52)	237 (41.80)	
**Family history of DM**				
No	312 (76.47)	96 (23.53)	408 (71.96)	0.017
Yes	136 (85.53)	23 (14.47)	159 (28.04)	
**Type of DM**				
T1DM	148 (93.08)	11 (6.92)	159 (28.04)	0.000
T2DM	300 (73.53)	108 (26.47)	408 (71.96)	
**Type of treatment**				
OHA	229 (74.35)	79 (25.65)	308 (54.32)	0.000
Insulin	149 (92.55)	12 (7.45)	161 (28.40)	
Both	70 (71.43)	28 (28.57)	98 (17.28)	
**History of AKI**				
No	359 (82.53)	76 (17.47)	435 (76.72)	0.000
Yes	89 (67.42)	43 (32.58)	132 (23.28)	
**Proteinuria**				
Negative	365 (82.77)	76 (17.23)	441 (77.78)	0.000
Positive	83 (65.87)	43 (34.13)	126 (22.22)	
**Hypertension**				
No	258 (87.76)	36 (12.24)	294 (51.85)	0.000
Yes	190 (69.60)	83 (30.40)	273 (48.15)	
**Diabetic retinopathy**				
No	382 (84.33)	71 (15.67)	453 (79.89)	0.000
Yes	66 (57.89)	48 (42.11)	114 (20.11)	
**Cardiovascular disease**				
No	398 (81.56)	90 (18.44)	488 (86.07)	0.000
Yes	50 (63.29)	29 (36.71)	79 (13.93)	
**Nephropathy**				
No	391 (81.63)	88 (18.37)	479 (84.48)	0.000
Yes	57 (64.77)	31 (35.23)	88 (15.52)	

### Physiologic characteristics

Physiological characteristics and comparisons based on DPN status during follow-up are presented in Table 2. More incident DPN was observed in those obese DM patients at baseline. Similarly, those with greater baseline TC, TG, LDL-C, SBP, DBP, and lower HDL-C levels, as well as lower HDL-C levels, had a larger percentage of new DPN.

**Table 2:** Baseline physiologic characteristics of study participants by incident diabetic peripheral neuropathy status during of follow-up from 2013 to 2021

Variables	Incident Peripheral Neuropathy		Total (n = 567)	P-value
	No DPN (n = 448)	DPN (119)		
**Total cholesterol(mg/dl)**				
Desirable	302 (84.12)	57 (15.88)	359 (63.54)	0.000
Borderline	83 (76.85)	25 (23.15)	108 (19.12)	
High	61 (62.24)	37 (37.76)	98 (17.35)	
**Triglyceride(md/dl)**				
Normal	240 (84.21)	45 (15.79)	285 (50.26)	0.001
Borderline	94 (79.66)	24 (20.34)	118 (20.81)	
High	114 (69.51)	50 (30.49)	164 (28.92)	
**LDL-C(md/dl)**				
<100	291 (83.14)	59 (16.86)	350 (61.73)	
>=100	157 (72.35)	60 (27.65)	217 (38.27)	
**HDL-C(md/dl)**				
>=40	353 (81.34)	81 (18.66)	434 (76.54)	0.014
<40	95 (71.43)	38 (28.57)	133 (23.46)	
**BMI (kg/m** ^**2**^ **)**				
Underweight	35 (89.74)	4 (10.26)	39 (6.88)	0.000
Normal	311 (87.11)	46 (12.89)	357 (62.96)	
Overweight	80 (62.50)	48 (37.50)	128 (22.57)	
Obese	22 (51.16)	21 (48.84)	43 (7.58)	
**SBP**				
<140	305 (84.25)	57 (15.75)	362 (63.84)	0.000
>/=140	143 (69.76)	62 (30.24)	205 (36.16)	
**DBP**				
<90	323 (82.61)	68 (17.39)	391 (68.96)	0.002
>/=90	125 (71.02)	51 (28.98)	176 (31.04)	

**Abbreviation-** BMI; body mass index, DBP; diastolic blood pressure, HDL-C; high density lipoprotein cholesterol, LDL-C; low density lipoprotein cholesterol, SBP; systolic blood pressure

*Note that: Total cholesterol(mg/dl) (Desirable- [< 200], borderline- [200–239], High- [≥240]), Triglyceride(mg/dl) (normal- [<150], borderline- [150–199], High- [≥200])*

**Table 3:** Model comparison using Akaike and Bayesian information criteria

Model	AIC	BIC
Cox	1357.824	1405.529
Exponential	689.0384	741.0803
Weibull	669.9168	726.2955
Gompertz	662.7615	719.1403

**Abbreviation-** AIC; Akaike information criteria, BIC; Bayesian information criteria

**Table 4:** Multivariable Gompertz proportional hazard model results for predictors of incident diabetic peripheral neuropathy among diabetes mellitus patients, 2021

Variables	Categories	CHR [95% CI]	AHR [95% CI]
**Age**	< 65	1	1
	>/=65	2.02 [1.28, 3.18]	1.25 [0.76, 2.06]
**Sex**	Male	1	1
	Female	1.31 [0.91, 1.88]	1.47 [1.01, 2.15] **
**Type of DM**	T1DM	1	1
	T2DM	4.44 [2.39, 8.26]	3.49 [1.82, 6.71] ***
**Nephropathy**	No	1	1
	Yes	1.94 [1.29, 2.92]	1.56 [0.30, 2.05]
**DR**	No	1	1
	Yes	2.70 [1.87, 3.90]	1.90 [1.25, 2.91] ***
**SBP**	< 140	1	1
	>/=140	1.99 [1.39, 2.86]	1.02 [0.68, 1.54]
**Total cholesterol**	Desirable	1	1
	Borderline	1.40 [0.88, 2.24]	1.03 [0.60, 1.77]
	High	2.60 [1.72, 3.93]	1.58 [0.93, 2.69]
**Proteinuria**	Negative	1	1
	Positive	2.01 [1.38, 2.92]	**2.22 [1.35, 3.65]** ***
**LDL**	< 100	1	1
	>=100	1.61 [1.13, 2.31]	1.02 [0.66, 1.56]
**BMI**	Underweight	1	1
	Normal	1.39 [0.50, 3.87]	1.49 [0.52, 4.29]
	Overweight	4.28 [1.54, 11.86]	3.34 [1.09, 10.25] **
	Obese	5.29 [1.81, 15.41]	3.94 [1.20, 12.89] ***
**Triglyceride**	Normal	1	1
	Borderline	1.29 [0.78, 2.11]	0.71 [0.40, 1.24]
	High	1.95 [1.30, 2.91]	0.79 [0.47, 1.32]

**Abbreviation-** AHR; Adjusted hazard ratio, BMI; body mass index, CRH; Crude hazard ratio, DBP; diastolic blood pressure, DM; Diabetes mellitus, DR; Diabetic retinopathy, LDL-C; low density lipoprotein cholesterol, SBP; systolic blood pressure

** indicates P-value < 0.01 and *** P-value < 0

### Incidence of DPN and survival probability

After diagnosis T1DM and T2DM, subjects were followed for a total of 3174.78 PY, with a median follow-up period of 5.79 years (minimum of.09 and maximum of 7.48 years). The cumulative incidence of DPN was 21% (95% CI: [17.82, 24.55]). The total incidence rate was found to be 3.75, with a 95%CI of [3.13, 4.49] per 100 PY. T1DM had an incidence rate of 1.14; 95% CI: [0.63, 2.06] and T2DM had an incidence rate of 4.89; 95% CI: [4.04, 5.90] per 100 PY.

**Fig. 2:**
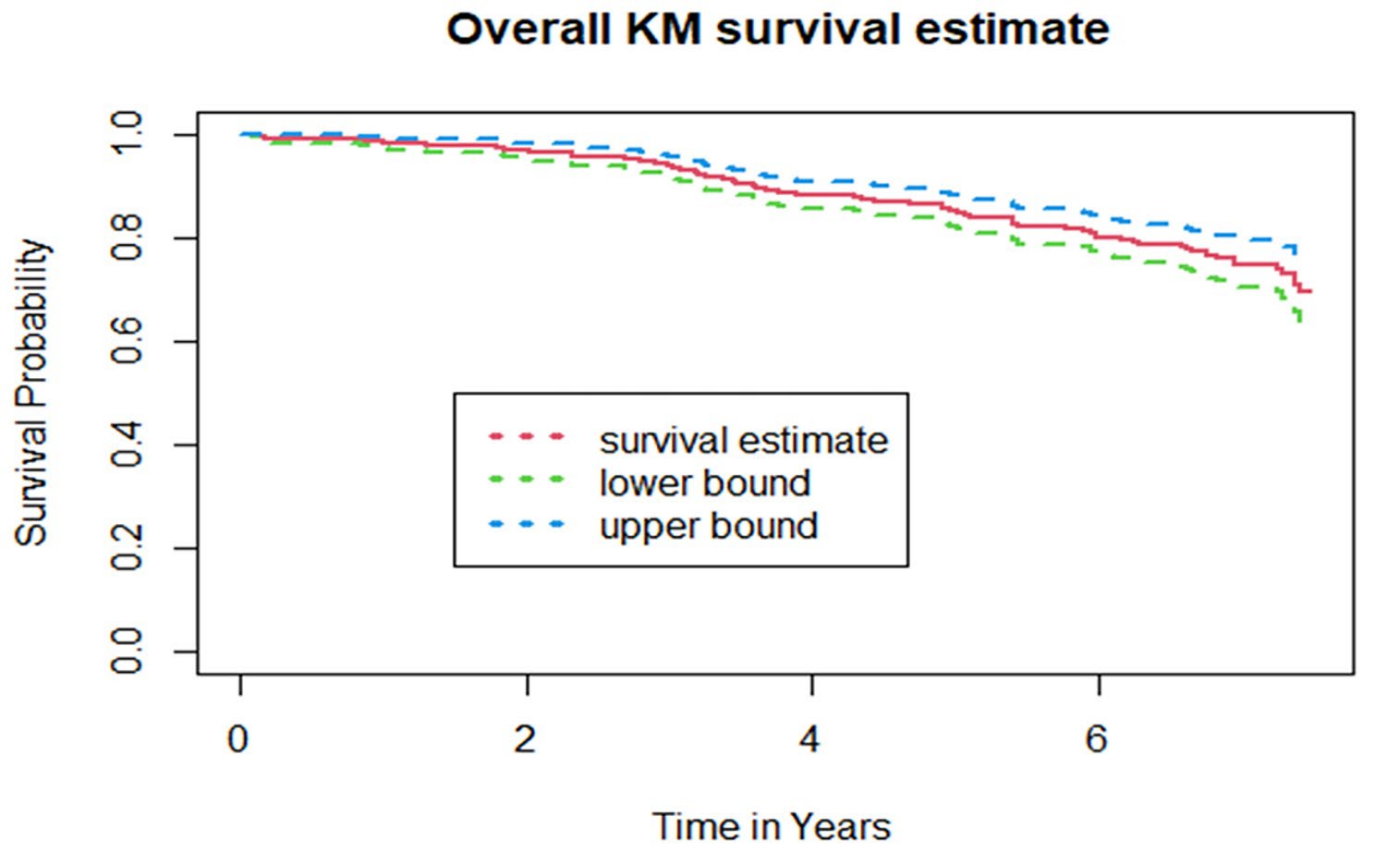
Overall Kaplan Meier of survival curves for diabetes mellitus patients on treatment at tertiary health-care setting of Southwest Ethiopia, from 2013 to 2021

**Fig. 3:**
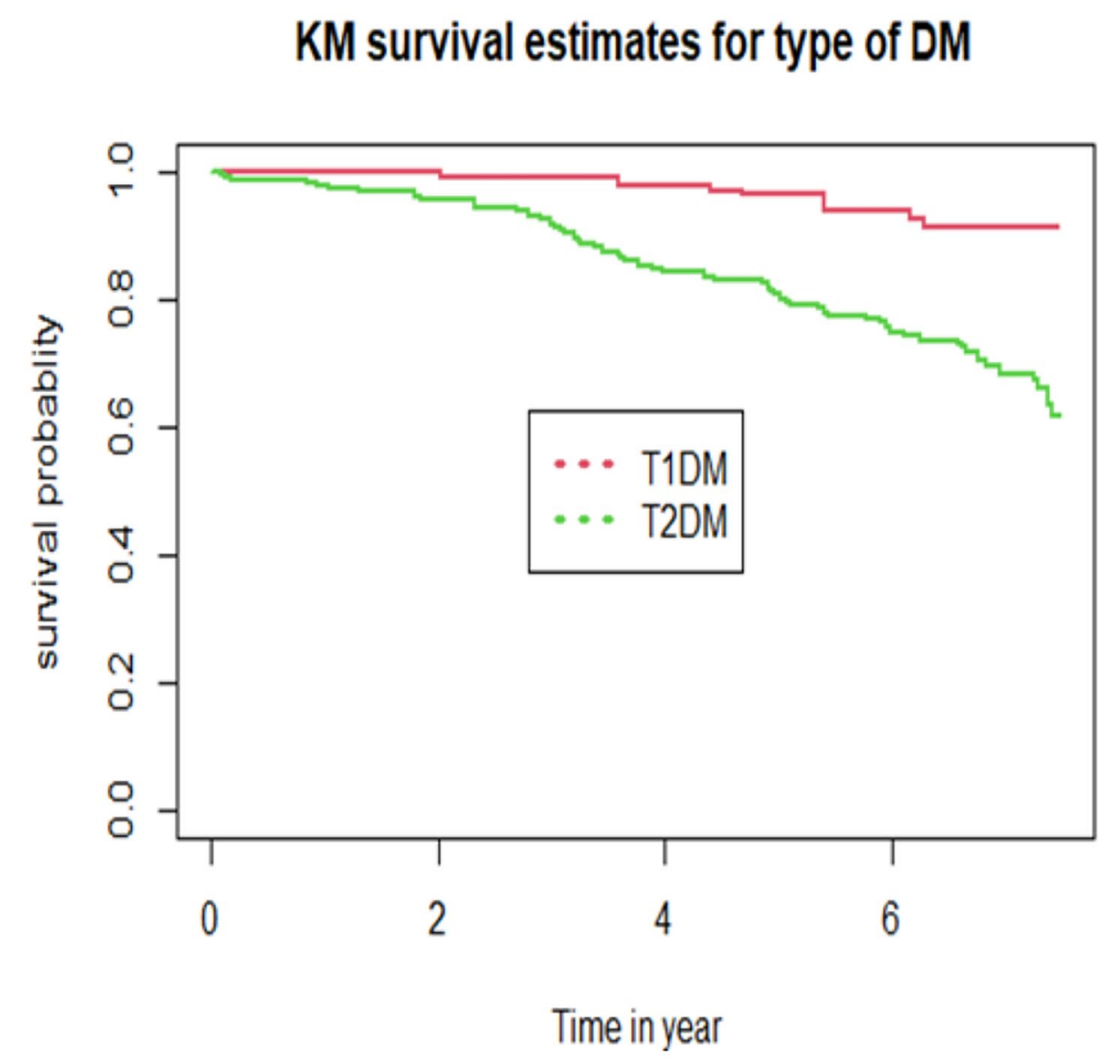
Kaplan Meir survival curves by type of diabetes mellitus patients on treatment at on treatment at tertiary health-care setting of Southwest Ethiopia, from 2013 to 2021

**Fig. 4:**
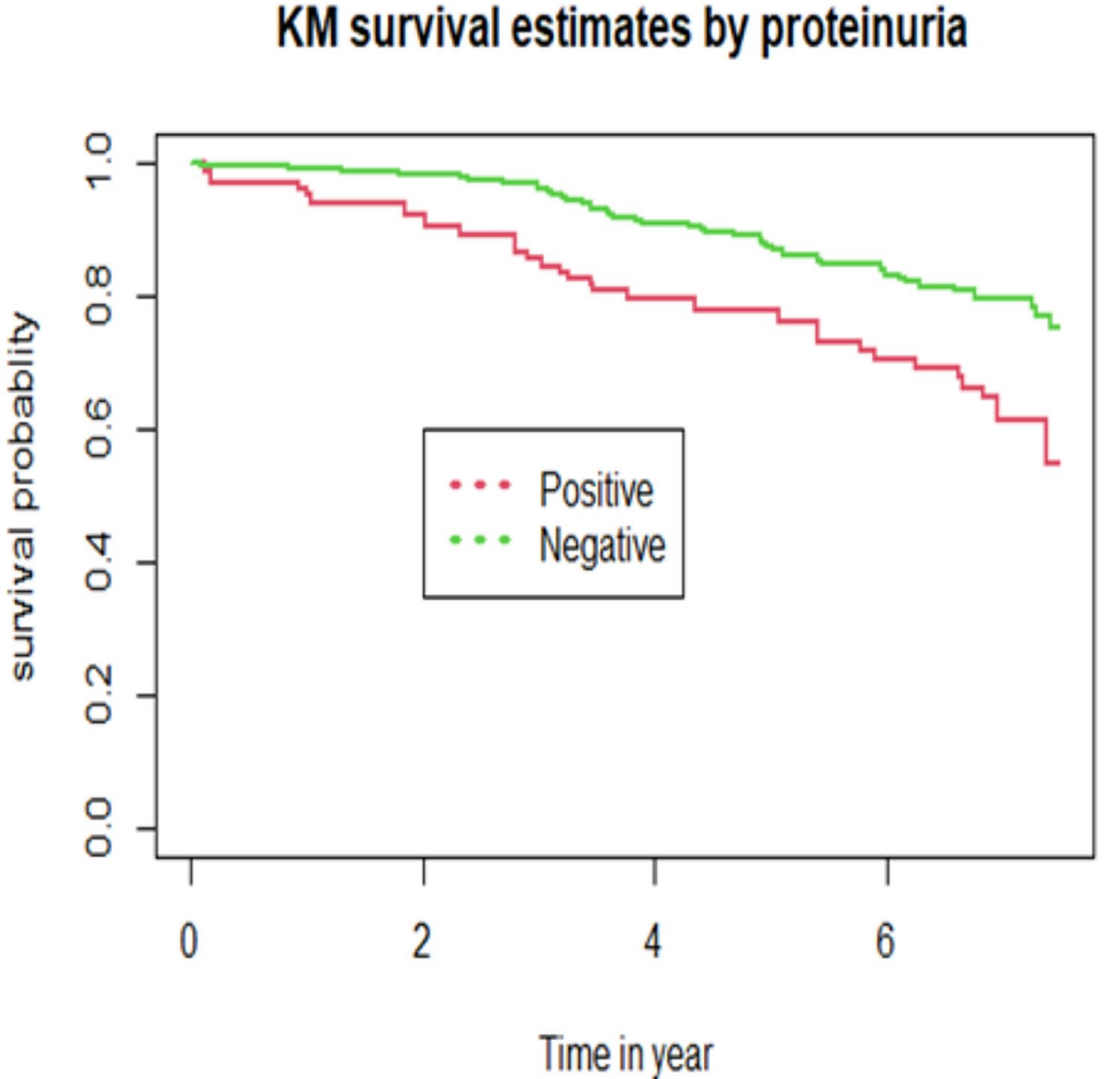
Kaplan Meir survival curves by proteinuria status for diabetes mellitus patients on treatment at on treatment at tertiary health-care setting of Southwest Ethiopia, from 2013 to 2021

The cumulative probability of event free survival was found to be 0.966, 0.878, 0.80 and 0.696 at 2-year, 4-year, 6-year and the end of the follow-up period, respectively (Fig. 2). Because the study ended before half of the participants developed DPN, the median survival time was not met. Furthermore, a Kaplan–Meier survival estimate revealed that individuals with T2DM (Fig. 3) and positive proteinuria (Fig. 4) at baseline had an increased risk of developing DPN than their counterparts.

### Predictors of diabetic peripheral neuropathy

As variable selection precedes model diagnostics, 11 factors significantly associated with incident DPN in the univariate analysis at p values less than 0.2 were included in the multivariable survival model. We used the Gompertz proportional hazard model, which has the lowest AIC when compared to other models, to examine the predictors of new-onset DPN (Table 3). The pseudo VIF values ranged from 1.04 to 1.62, showing that the independent variables were not multicollinear. PHA was met because the Schoenfeld residual to test the PHA was not significant (P-values for each variable ranged from 0.462 to 0.949, with a global P-value of 0.624). Furthermore, the Cox Snell residual plot revealed that the model’s goodness of fit was fulfilled because the cumulative hazard plot follows a 45 degree or a straight line through the origin with slope one. In the multivariable Gompertz proportional hazard model, there are five independent variables (female sex, positive proteinuria, DR, T2DM, and high BMI) that significantly predicts the likelihood of DPN in diabetic patients after controlled for other variables (Table 4).

Keeping other variables constant, the hazard of developing DPN for female was 47% higher than males [AHR = 1.47; 95% CI (1.01, 2.15)]. Similarly, the hazard of DPN was increased by 3.49 among newly diagnosed T2DM [AHR = 3.49 95% CI (1.82, 6.71)] compared with T1DM. Holding other variables constant, the risk of developing DPN was increased by 1.90 [AHR = 1.9 95% CI (1.25, 2.91)] and 2.22 [AHR = 2.22 95% CI (1.35, 3.65)] among participants who had DR and proteinuria respectively. Adjusting for other variables, the risk of developing DPN was increased by 3.34 [AHR = 3.34 95% CI (1.09, 1.025)] and 3.94 [AHR = 3.94 95% CI (1.2, 12.89)] times among obese and overweight when compared to their counter parts respectively.

## Discussion

DPN is a global life-threatening disease that has a significant impact on patients’ quality of life which present a sizable financial burden to patients and health care system. Thus, this study determined the incidence and predictors of DPN among newly diagnosed T1DM and T2DM patients in tertiary health care setting of southwest Ethiopia. A total of five factors were identified to significantly predict the incidence of DPN and these factors include: Female sex, T2DM, DR, positive proteinuria, and high BMI.

The findings of this study showed that the cumulative incidence of DPN was 21% [17.82, 24.55] while incidence rate was 3.75, with a 95%CI of [3.13, 4.49] per 100 PY. The incidence of the current study is in line with a prospective cohort study conducted in Austria [7]. However, the finding is higher than studies conducted in Denmark and North West Ethiopia which was 10% and 16.63%, respectively [21, 29]. This discrepancy might be due to the different populations included in those studies and shorter follow up compared with our study for those conducted in Denmark. The other possible reason could be due to higher percentage of obesity and overweight in our study population compared to those conducted in North west Ethiopia. The incidence of DPN in the current study is lower than previous studies conducted in Jordan 39.5%, in Germany 40.3%, and 29.4% in Uganda [11, 33, 34]. This might be owing to the fact that, typically, DPN is often diagnosed clinically with little further laboratory investigations to confirm the diagnosis or poorly captured in patients’ records [35]. In a study by Day et al. [36], more than 40% of diabetic patients in general practice had no biochemical evaluation. The wide geographical variations in incidence of DPN highlight the need for a global multicenter study in which the selection criteria for diabetic patients are unified to the finest detail.

According to this study, being female significantly predicts the risk of DPN. The hazard of developing DPN was increased by 47% among females compared with males. This result agrees with studies done in Jordan and Japan which showed that the female has about six times more likely to develop DPN compared with male [37, 38]. However, The results of the study are in stark contrast to those of most studies in the literature [39, 40] whereas another study found no significant association and variation in gender [41]. This discrepancy may be a result of differences in the tests used to measure neuropathic changes, the duration of diabetes, and the level of glycemic control for the subjects. These inconsistent findings might show that further high-standard research is needed to put evidence on the association between gender and developing DPN.

The risk of developing DPN was twice among participants who had positive proteinuria than those with negative proteinuria. Albuminuria has been proposed to be an independent predictor of increasing levels of microvascular and macrovascular diseases in DM patients [42]. However, the number of published studies with the specific aim of investigating the correlation between albuminuria and DPN are very limited, although the results of some studies from Iran [43] and Taiwan [44] and Vietnam [45] may indirectly reflect this association. High serum cystatin C levels were linked to DPN in Chinese research [46], suggesting that this molecule could be used as a biomarker for DPN in DM patients. Callaghan et al. [47] suggested in their review that neuropathy can be particularly severe in diabetic chronic kidney disease. To date, the mechanisms of neurotoxicity in DM patients with renal impairment due to proteinuria remains unclear. However, the experimental evidence indicates that renal impairment result in alteration in membrane excitability which consequently, leading to an accumulation of extracellular K + that causes depolarization [48]. Disruption of these various ionic gradients may affect the Na+/Ca2 + exchanger, leading to increased levels of intracellular Ca2 + and axonal loss [49]. In addition, previous research as impaired renal function results in endothelial injury [50] which eventually, leads to neuropathy due to impaired nerve blood flow, and reduced nerve oxygen tension.

According to the findings, the hazards of developing DPN were 90% higher for those participants with DR at baseline compared with their counterparts. This finding is in line with the previous studies conducted in Germany, Libya and Vietnam which showed that the likelihood of DPN was almost twice among patients with DR [11, 45, 51]. A meta-analysis strongly supported the significant association between DPN and DR by showing a similar increase in risk of developing DPN in the patients who have complications [52]. The coexistence of diverse diabetic problems could be explained by the fact that the majority of diabetic complications have comparable risk factors, particularly poor glycemic control. The other reason could be that both complications have the same pathophysiology, and hence the existence of one complication could indicate the presence of the other. Diabetes-induced hyperglycemia has a role in the pathogenesis of DPN and retinopathy, with a link between the buildup of advanced glycation end products and the activation of the polyol pathway, protein kinase C, and free radicals, resulting in biomolecular damage in the affected tissues [53].

Having T2DM increases the hazard of DPN by more than 3-fold when compared to T1DM participants. This finding is consistent with the cohort study conducted among youth in the USA which showed youth with T2DM were four times more likely to develop DPN compared with T1DM [22, 54]. Similarly, other systemic review study showed a higher percentage of T2DM patients with symptoms of neuropathy compared with T1DM patients [55]. The possible explanation for this could be that sensory nervous impairment occurs earlier in type 2 diabetes than in T1DM [56]. This could be due to the physiology of the somatosensory pathways, and it’s interesting to note that, while traveling through separate sensory pathways, both superficial and deep sensitivity mainly degrade in T2DM [57]. The other possible reason could be the currently decreasing age of onset of T2DM allowing enough time for the development of DM complication [58].

The BMI is a standard unit for quantifying overweight and obesity. DPN was also more in overweight and obese than in nonobese/normal cases ‑ 48.8%, 37.5% and 12.9%, respectively. Results of the current study also supports this evidence as being obese and overweight increases the hazard of DPN by approximately four and three folds, respectively. This is in line with previous studies that revealed the obese group was more likely to have DPN than the non-obese group [22, 59]. Five recent epidemiological studies two from China [60, 61], and one from Denmark [21], one from Netherlands [62] and one from Germany [63] also implicate obesity as the second most influential metabolic risk factor for neuropathy after diabetes. The possible explanation for this might be due to high risk of overweight and obesity for different chronic conditions in human beings which consistently applicable in developing DPN.

## Conclusion and recommendation

The incidence of DPN was relatively high compared with previous studies. Nearly, 7 in 10 of DPN cases occurred within a short period (5 years) of DM diagnosis. Being female, T2DM, DR, positive proteinuria, being obese and overweight significantly predicts the risk of DPN. Therefore, we recommend screening and early diagnosis of diabetes and its complications. Given the significancy of obesity as a DPN risk factor, educating DM patients on a healthy lifestyle and the benefit of regular checkups to reduce obesity is recommended. While doing so, attention should be given for DM patients with DR and positive proteinuria. Further prospective study on the topic is recommended by including some missed variables. Since female sex is contradictory whether used as a protective or risk for DPN in DM patient, we recommend further research on this issue.

### Limitation of the study

In spite of its strength, we acknowledge several limitations in our study. The nature of study design as a retrospective study limits the accurate assessment of the association between DPN and risk factors. Due to the retrospective nature of data, we have not considered other potential predictors such as exercise, adherence to treatment, smoking, alcohol drinking and diet due to their unavailability in the patients’ card. Study assumed all the DPN were caused by DM and relying only on clinical signs and symptoms to confirm the diagnosis of DPN may underestimate its incidence in the study.

## Data Availability

Data will be available from the corresponding author upon request.

## Abbreviations

AKI: Acute Kidney Injury
AHR: Adjusted Hazard Ratio
AIC: Akakie Information Criterion
BMI: Body Mass Index
CVD: Cardiovascular Disease
DM: Diabetes Mellitus
DPN: Diabetic Peripheral Neuropathy
DR: Diabetic Retinopathy
HDL-C: High Density Lipoprotein Cholesterol
IDF: International Diabetic Federation
HTN: Hypertension
JUMC: Jimma University Medical center
LDL-C: Low Density Lipoprotein Cholesterol
MKCSH: Mettu Karl Comprehensive and Specialized Hospital
PHA: Proportional Hazard Assumptions
PY: Person Years
T1DM: Type one DM
T2DM: Type two DM
